# Positive contrast high-resolution 3D-cine imaging of the cardiovascular system in small animals using a UTE sequence and iron nanoparticles at 4.7, 7 and 9.4 T

**DOI:** 10.1186/s12968-015-0167-4

**Published:** 2015-07-07

**Authors:** Aurélien J. Trotier, William Lefrançois, Kris Van Renterghem, Jean-michel Franconi, Eric Thiaudière, Sylvain Miraux

**Affiliations:** Centre de Résonance Magnétique des Systèmes Biologiques, UMR 5536 CNRS/Université de Bordeaux, 146 rue Léo Saignat, Cedex 33076 Bordeaux, France

**Keywords:** UTE (Ultra-short Echo Time), Iron based nanoparticles, Positive contrast, Cine 3D, Mouse models, Cardio-vascular

## Abstract

**Background:**

To show that 3D sequences with ultra-short echo times (UTEs) can generate a positive contrast whatever the magnetic field (4.7, 7 or 9.4 T) and whatever Ultra Small Particles of Iron Oxide (USPIO) concentration injected and to use it for 3D time-resolved imaging of the murine cardiovascular system with high spatial and temporal resolutions.

**Methods:**

Three different concentrations (50, 200 and 500 μmol Fe/kg) of USPIO were injected in mice and static images of the middle part of the animals were acquired at 4.7, 7 and 9.4 T pre and post-contrast with UTE (TE/TR = 0.05/4.5 ms) sequences. Signal-to-Noise Ratio (SNR) and Contrast-to-Noise Ratio (CNR) of blood and static tissus were evaluated before and after contrast agent injection. 3D-cine images (TE/TR = 0.05/3.5 ms, scan time < 12 min) at 156 μm isotropic resolution of the mouse cardiopulmonary system were acquired prospectively with the UTE sequence for the three magnetic fields and with an USPIO dose of 200 μmol Fe/kg. SNR, CNR and signal homogeneity of blood were measured. High spatial (104 μm) or temporal (3.5 ms) resolution 3D-cine imaging (scan time < 35 min) isotropic resolution were also performed at 7 T with a new sequence encoding scheme.

**Results:**

UTE imaging generated positive contrast and higher SNR and CNR whatever the magnetic field and the USPIO concentration used compared to pre-contrast images. Time-resolved 3D acquisition enables high blood SNR (66.6 ± 4.5 at 7 T) and CNR (33.2 ± 4.2 at 7 T) without flow or motion artefact. Coronary arteries and aortic valve were visible on images acquired at 104 μm resolution.

**Conclusions:**

We have demonstrated that by combining the injection of iron nanoparticles with 3D-cine UTE sequences, it was possible to generate a strong positive contrast between blood and surrounding tissues. These properties were exploited to produce images of the cardiovascular system in small animals at high magnetic fields with a high spatial and temporal resolution. This approach might be useful to measure the functional cardiac parameters or to assess anatomical modifications to the blood vessels in cardio-vascular disease models.

**Electronic supplementary material:**

The online version of this article (doi:10.1186/s12968-015-0167-4) contains supplementary material, which is available to authorized users.

## Background

In clinical practice, magnetic resonance angiography (MRA) enhanced by a gadolinium-based contrast agent is the gold standard for many applications [1–3] (MRA of the lower limbs, supra-aortic MRA, dynamic MRA, etc.).

However, the use of this type of contrast agent has two major disadvantages: 1) intra-venous injection entails a risk for the patient to develop nephrogenic systemic fibrosis [4]; 2) first pass extraction and rapid redistribution into the extracellular space limits the time-window of imaging enhanced vasculature [5]. One way to overcome this limitation is to use contrast agents with higher blood half-life. Some gadolinium-based contrast agents have been developed and validated in pre-clinical models [6].

The use of ultrasmall superparamagnetic iron oxides (USPIOs) can also appear as a good alternative [7, 8]. In patients, numerous studies at 1.5 T described the use of USPIO-based contrast agents for contrast-enhanced angiography [5, 9] and coronary MR angiography [10]. Similar positive contrast was also obtained at low field strength (0.5 T) [11]. Recently, ferumoxytol was used at 3 T for contrast-enhanced high resolution imaging of the cardio-vascular system [12, 13].

In small animals, the use of USPIO-type contrast agents is even more common, but is rarely applied to vascular imaging. In fact, they are mostly used for cell tracking [14, 15] or molecular imaging [16–19]. In these domains, and for reasons of sensitivity, they are rather used as T2* negative contrast agents and at high field strength. Nevertheless, several methods, like off-resonance saturation (ORS) techniques [20–22], have been developed to provide a positive contrast with USPIO-type agents.

These methods use the disturbances of the magnetic field induced by USPIOs. Their limitation, however, is that such disturbances can arise from other sources (air/tissue interface, imperfections in B0 field homogeneity, etc.). Furthermore, these effects are even more prevalent at high magnetic fields. They may prevent the use of these methods for highly resolved angiography at high magnetic fields commonly used in preclinical studies.

Iron-based contrast agents can produce a positive effect at high fields for angiography if the T2 and T2* effects are limited, and their slight T1 effect availed of. To do this, it is necessary to limit the signal decay caused by the phase-shifts of the spins surrounding the USPIOs. This phenomenon is enhanced by significant and turbulent blood flows in some blood vessels. The best way to limit this signal decay is to drastically reduce the echo times (TEs) of the MRI sequences.

Ultrashort TE (UTE) pulse sequences allow for signal acquisition with little T2 influence [23] and have been used to probe the T1 effect generated by USPIO. This approach has shown great promise for in vivo applications particularly in the field of quantitative imaging [24, 25].

The aim of this study was to show that 3D imaging with ultra-short echo times (TE < 0.050 ms) can generate a positive contrast for blood other a wide range of magnetic fields (4.7, 7 or 9.4 T) and USPIO concentrations injected. This method was combined to a new 3D cine UTE encoding scheme, to provide 3D time-resolved images of the murine cardiovascular system with either very high spatial or very high temporal resolutions.

## Methods

### Magnets and gradient systems

Experiments were performed on 4.7, 7 and 9.4-Tesla Bruker Biospec Systems (Ettlingen, Germany) equipped with gradient systems capable of 660 mT/m maximum strength and 110-μs rise time. Two different coil systems were used, depending on experiments: (a) a mouse-dedicated probe (birdcage resonator, 35 mm in diameter and 60 mm long at 4.7 T (static images), 7 T (static images) and 9.4 T (static and cine images); (b) a volume resonator (75.4 mm inner diameter, active length 70 mm) operating in quadrature mode was used for transmission, and a four-element (2 × 2) phased array surface coil (outer dimensions of one coil element: 12 × 16 mm^2^; total outer dimensions: 26 × 21 mm^2^) was used for signal reception at 4.7 and 7 T for cine imaging.

### *In-vivo* MR experiments

#### Contrast agents

Three concentrations of Sinerem contrast agent (Guerbet, Aulnay-sous-bois, France) were used: 50 μmol Fe/kg - 2.8 mg Fe/kg, 200 μmol Fe/kg - 11.2 mg Fe/kg, and 500 μmol Fe/kg - 28 mg Fe/kg. The r1 and r2 for Sinerem measured in saline were 1.14 ± 0.06 mM^−1^.sec^−1^ and 36.46 ± 3.03 mM^−1^.sec^−1^ at 4.7 T, 1.13 ± 0.09 mM^−1^.sec^−1^ and 65.21 ± 4.23 mM^−1^.sec^−1^ at 7 T and 1.14 ± 0.1 mM^−1^.sec^−1^ and 86.23 ± 3.81 mM^−1^.sec^−1^ at 9.4 T, respectively.

### Animal preparation

All experimental procedures were approved by the Institutional Ethics committee for Animal Care and Use at Bordeaux university, France (Approval No. 5012032-A).

### Static imaging

Mice (C57BL/6, *n* = 3 for each condition, body weights: 21-25 g) were anesthetized with isoflurane (1.0 % in air) and 100 μL of contrast agent was injected through the tail vein. Imaging was performed before and after injection of the contrast agent; total imaging duration was 1 h.

### Cine imaging

Mice (C57BL/ 6, *n* = 12, body weights: 21–25 g) were anesthetized with isoflurane (1.0 % in air). The ECG signal was picked up using electrodes wrapped around the forelimbs. This signal was converted into a square trigger pulse by a specific monitoring and gating system (SA Instruments, Inc., NY, USA) connected to the spectrometer. A respiratory sensor was placed under the animal’s thorax. ECG and respiratory signals were visualized on a user-interface; cardiac rhythm was stabilized (380–420 beats/min) and anesthesia was regulated by modifying the proportion of isoflurane inhaled. A 100-μL volume of 200 μmol Fe/kg was injected through the tail vein. Images were acquired pre and post injection of the contrast agents.

### MRI parameters

#### Static imaging

To determine the signal-to-noise ratio and contrast-to-noise ratio obtained before and after injection of the three USPIO concentrations at 4.7, 7 and 9.4 T, non-synchronized images were acquired with 3D UTE sequences on a large field-of-view (FOV) (extending from liver to neck). Imaging parameters are indicated in Table 1.

**Table 1:** Imaging parameters used for static and cine images

Static imaging		Cine-imaging	
Imaging parameters	UTE	Mid resolution UTE	High resolution UTE
Coil/Magnetic Field	Volumetric/ 4.7, 7, 9.4 T	Volumetric/9.4 T phased array/ 4.7, 7 T	phased array/7 T
TR/TE (ms)	4.5/0.031	3.5/0.031	3.5/0.031
Excitation Pulse/ duration (ms) / FA°	square / 0.05 / 15°	square / 0.05 / 15°	square / 0.05 / 15°
Field of view (mm)	30 × 30 × 30	20 × 20 × 20	20 × 20 × 20
Matrix	128 × 128 × 128	128 × 128 × 128	192 × 192 × 192
Number of projections	51360	18144/cine	52540/cine
Resolution (μm)	234 × 234 × 234	156 × 156 × 156	104 × 104 × 104
Bandwith (Hz/Pixel)	781	781	520
Triggering	No	ECG	ECG
Cine images (N)	-	10	10
Excitations (N)	1	1	1
Total acquisition time	3 min 51 s	11 min 20 s	32 min 50 s

### Cine imaging

3D cine UTE images were acquired at 4.7, 7 and 9.4 T with an isotropic resolution of 156 μm, hereafter called “mid-resolution UTE” and at 7 T with an isotropic resolution of 104 μm, hereafter called “high-resolution UTE”.

**Fig. 1:**
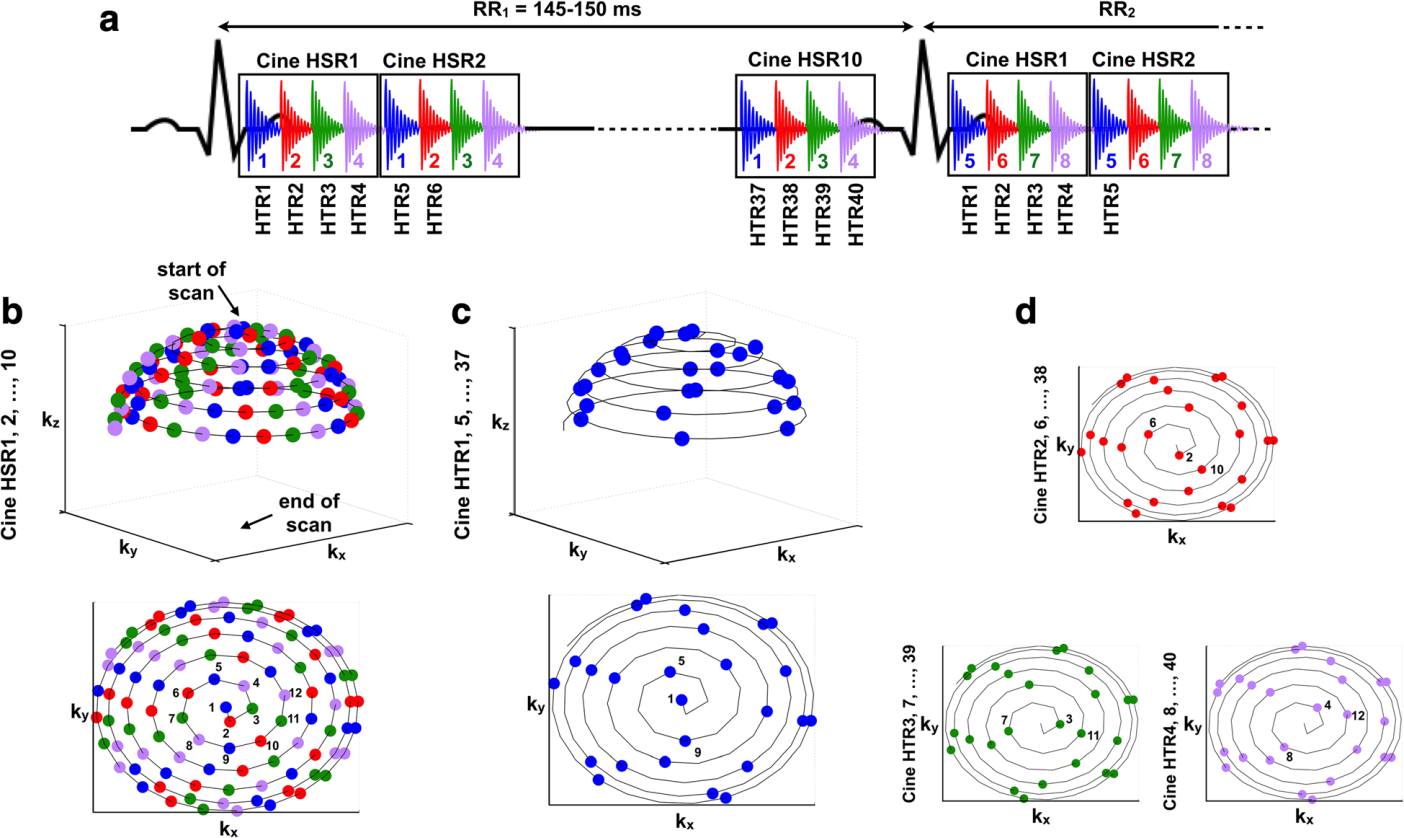
Acquisition scheme of the 3D ECG-triggered UTE sequence used to reconstruct either 10 High Spatial Resolution cine images or 40 High Temporal Resolution cine images. **a** Blocks of 4 Free Induction Decays (FIDs) corresponding to 4 k-space trajectories (named « 1-2-3-4 » for the first R-R interval, « 5-6-7-8 » for the second R-R interval…) were repeatedly acquired 10 times along one R-R interval. **b** (*up*) Point surface showing the end-point of the acquired half-projections in a 3D k-space and (*bottom*) the corresponding projections on the k_x_-k_y_ plane, acquired 10 times for one R-R interval. The reconstructed images display high space resolution (HSR) and lower time resolution. **c** (*up*) Point surface showing the ends of the first acquired half-projection of each block in a 3D k-space and (*bottom*) the corresponding projections on the k_x_-k_y_ plane of the end-point of trajectories. The reconstructed images display higher time resolution (HR) at the expense of space-resolution. **d** Projections on the k_x_-k_y_ plane of the end-point of the second trajectories of each block used for HTR2, 6,… images in red, the third trajectories of each block used for HTR3, 7, … images in green and the fourth trajectories of each block used for HTR4, 8, … images in magenta

The acquisition scheme of the triggered prospective sequence is shown on Fig. 1. The proposed encoding scheme allows to reconstruct with the same acquisition datas, either Ncine = R-Rinterval/(4 x TR) High Spatial Resolution images (HSR), or Ncine = R-Rinterval/TR High Temporal Resolution (HTR) images.

Half-projections were sampled in a distribution previously described [26–28], with sampling starting at a pole of the sphere and spiraling down to the other pole as the scan progressed.

To reconstruct HSR images, block of 4 consecutive k-spaces trajectories (named « 1-2-3-4 » for the first R-R interval, « 5-6-7-8 » for the second R-R interval, …) were combined to describe a sphere with the maximum numbers of projections (52540 per cine). The same encoding was used for each cine HSR images (HSR1, HSR2, …, HSR10).

By using each projections per RR-interval individually, four-times more cine HTR images could be reconstructed with 4 times-less projections (13135). It corresponds to the sampling trajectory with the same color code described in Fig. 1c (blue). HTR1, HTR5, … HTR37 have the same sampling trajectory which slightly differs from the HTR2, HTR6, … HTR38 red trajectories, HTR3 to HTR 39 green trajectories and HTR4 to HTR40 magenta trajectories.

This method allows to homogeneously distribute projections on the surface of the sphere, both in HSR and HTR images.

Imaging parameters are indicated in Table 1.

### Reconstruction procedure

All the UTE data where reconstructed using the following procedure: k-space data were regridded with an oversampling ratio of 2 using a Kaiser-Bessel kernel [29]. Data were transformed by applying a conventional fast Fourier transform (FFT). Each phased array receiver magnitude image was reconstructed using the method described above, and then combined by a sum of squares reconstruction.

### Image analysis

#### Signal-to-noise ratio, contrast-to-noise ratio and signal homogeneity measurements

Igor Pro (Wavemetrics, Lake Oswego, OR) data processing software was used to calculate the apparent signal-to-noise ratio (SNR) for blood, myocardium and muscle. Apparent SNR was defined as the signal intensity for the Region Of Interest (ROI) divided by the standard deviation of the noise (measured for an ROI positioned outside the mouse body).

The contrast-to-noise ratio (CNR) for blood compared to stationary tissue was defined as CNR = SNR (blood) – SNR (myocardium or muscle). CNR was measured at the level of the aortic arch and jugular veins for static imaging, and in the ventricles for cine imaging.

Signal homogeneity of blood throughout the cycle was assessed using the measurements of the standard deviation for the blood signal in the left and right ventricles and in the aortic arch.

### Statistical analysis

Results were compared using an Anova-test. *P* < 0.05 was considered for a significant difference.

### Volume analysis

Volume analysis was performed on 6 mice with a semiautomated segmentation procedure on Amira (Visage Imaging GmbH, Germany) to calculate left ventricular end-diastolic volume, left ventricular end-systolic volume, left ventricular stroke volume (LVSV), left ventricular ejection fraction (LVEF), right ventricular end-diastolic volume, right ventricular end-systolic volume, right ventricular stroke volume (RVSV), and right ventricular ejection fraction (RVEF).

## Results

### Static imaging – pre and post contrast with UTE sequences

**Fig. 2:**
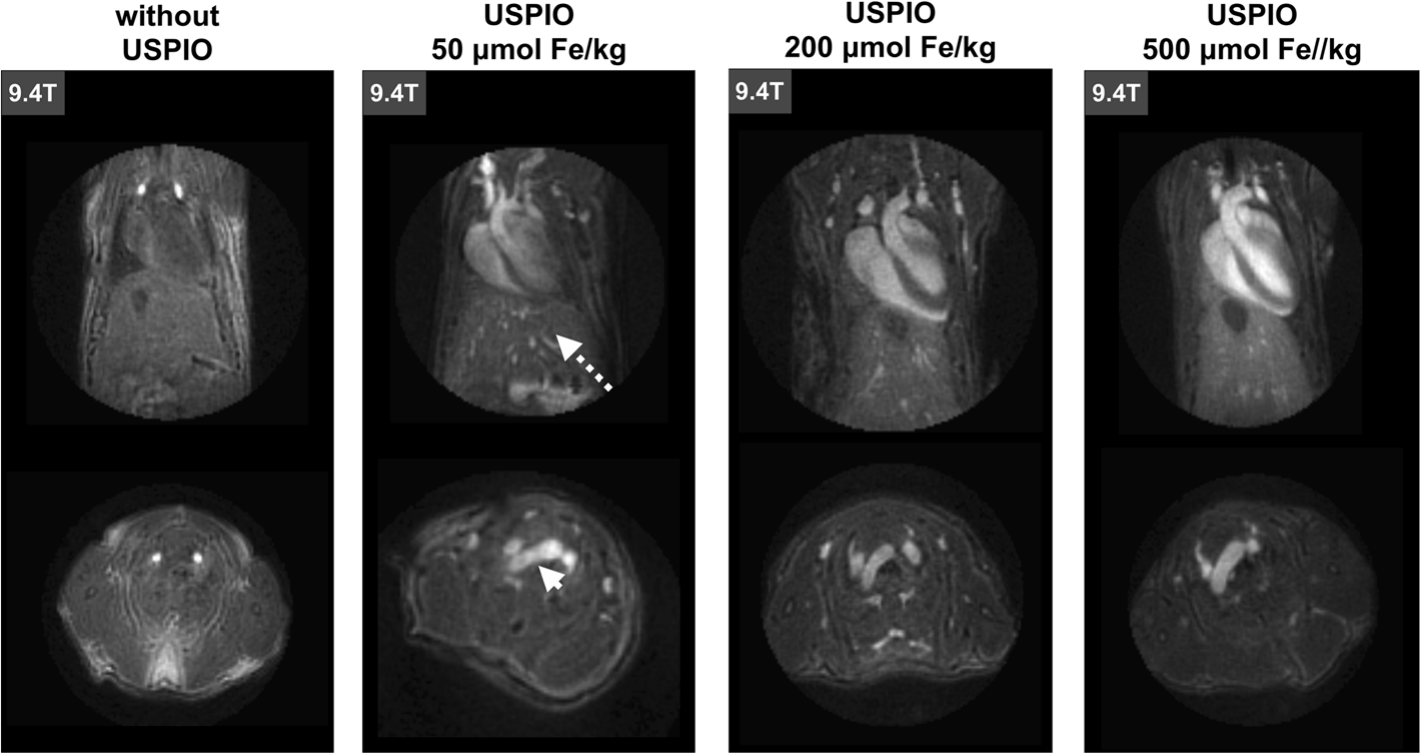
3D UTE images at 9.4 T showing the heart and the liver of a mouse. Images were acquired before and after injection of USPIO at 50, 200 and 500 μmol Fe/kg, without cardiac or respiratory synchronization. The small arrow indicates the aortic arch and the dashed arrow indicates the liver

Images were acquired in a zone extending from the liver to the neck. Three different concentrations of contrast agents were used, and the images acquired at 9.4 T pre- and post contrast agent injection with UTE sequences are shown in Fig. 2. Before injecting the contrast agent, the blood vessels and the blood inside the ventricles were not readily visible. After injection of the contrast agent, whatever the concentration injected, the blood in the various blood vessels was visible with the UTE sequence with an intense signal. This signal was highly homogeneous, whether in zones with high turbulence, such as the aortic arch (Fig. 2, small arrow), or in blood vessels with slow-flow, such as the jugular veins. No artefact related to blood flow or movement was visible on the images.

It should also be noted that the liver (Fig. 2, dashed arrow) always appears with a positive contrast and that its signal increases with USPIO concentration.

**Fig. 3:**
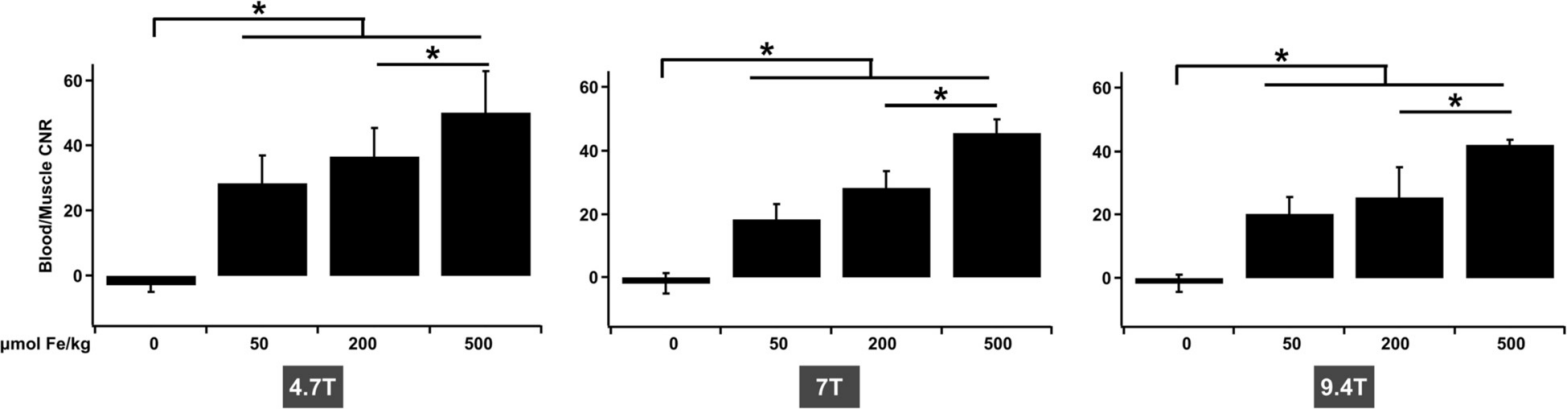
Contrast-to-noise ratio between blood in the aortic arch and muscle, obtained with UTE sequences before and after injection of USPIO at 4.7, 7 and 9.4 T and for different injection doses (200 and 500 μmol Fe/kg). p values lower than 0.05 are indicated by an asterisk

The same experiments were performed at 4.7 T, 7.0 T and 9.4 T, and the signal-to-noise ratio was measured for blood (aortic arch, jugular vein) and for the muscles. The blood-to-muscle contrast-to-noise ratios under the different conditions are indicated in Fig. 3.

Before contrast agent injection, contrast-to-noise ratio was very low (in the range of -2). After injection, whatever the magnetic field and the concentration of nanoparticles used, the contrast-to-noise ratio was always positive, and greater than or equal to 18. Increasing the concentration raised the contrast-to-noise ratio, at all magnetic fields (*p* < 0.05 for 200 μmol Fe/kg vs. 500 μmol Fe/kg). The highest concentration gave a better contrast-to-noise ratio, always greater than 40, whatever the magnetic field. Increasing the magnetic field slightly decreased the contrast-to-noise ratio, but this change was not significant.

It must be noted that for the lowest Sinerem concentration (50 μmol Fe/kg), the contrast varied slightly during the experiment (data not shown). This tended to decrease about 30 % over one hour with the UTE sequence. In contrast, the CNR measurements remained stable for at least one hour after injection for the two highest concentrations of contrast agent.

### Cine imaging

#### Mid-resolution UTE

**Fig. 4:**
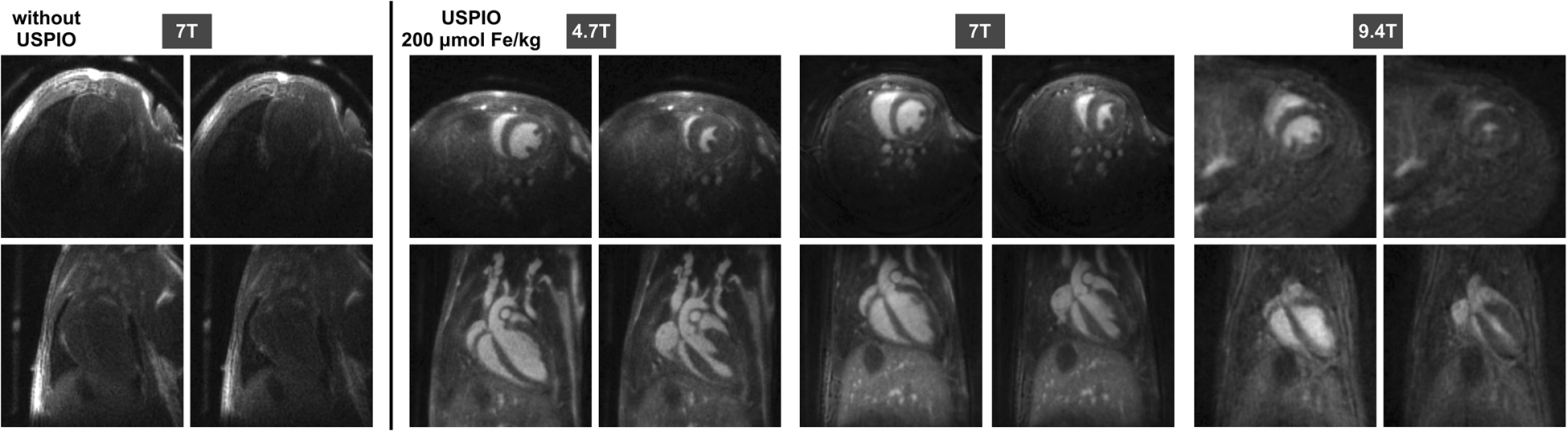
3D ECG-gated cine image of a mouse heart at 156 μm isotropic resolution obtained before (at 7 T) and after injection of USPIO at 200 μmol Fe/kg (at 4.7, 7 and 9.4 T). Ten images per cardiac cycle were generated, the images at the end of diastole (*left*) and systole (*right*) are shown in two orientations (*short axis: upper panels; long axis: lower panels*). No respiratory gating was used

3D-cine images at 156 μm isotropic resolution were acquired prospectively with the UTE sequence for the three magnetic fields tested before and after contrast agent injection. The intermediate concentration of contrast agent (200 μmol Fe/kg) was used for the images shown in Fig. 4, but similar results were obtained with the highest concentration. Slices were extracted during systole and diastole in two orientations (short axis and long axis) and are shown for the three magnetic fields. The acquisition times for these images were around 12 min.

Before injection, a contrast to noise ratio around -3 was obtained. After injection, and whatever the magnetic field, a positive contrast between myocardial blood and the myocardial wall was obtained at all times throughout the cardiac cycle (Table 2). The signal was also highly homogeneous, in both the blood vessels and in the heart cavities. For example, during a given experiment at 7 T, the standard deviation of the signal measured in the ventricles and the aortic arch during the cardiac cycle gave a value between 1.43 and 2.2 with an apparent SNR of 70.

**Table 2:** CNR values between the blood in the ventricles and the myocardial wall obtained with the UTE sequence at 156 μm isotropic resolution without USPIO and after injection of USPIO at 200 μmol Fe/kg

CNR (left ventricule - left myocardium)		End-diastole	End-systole
UTE 4.7 T	without USPIO	−2.6 ± 0.6	−4.0 ± 0.9
	USPIO 200 μmol Fe/kg	31.4 ± 3.8	30.2 ± 2.2
UTE 7 T	without USPIO	−2.5 ± 0.7	−3.9 ± 0.6
	USPIO 200 μmol Fe/kg	33.2 ± 4.2	29.2 ± 2.5
UTE 9.4 T	without USPIO	−2.1 ± 0.5	−3.9 ± 0.9
	USPIO 200 μmol Fe/kg	16.3 ± 5.8	15.2 ± 3.2

The images at 4.7 T and 7 T were of comparable quality, while at 9.4 T the signal-to-noise ratio and the contrast-to-noise ratio were lower. This discrepancy can be explained by the use of dedicated 4-channel receiver coils at 4.7 and 7 T only.

### High-resolution UTE

**Fig. 5:**
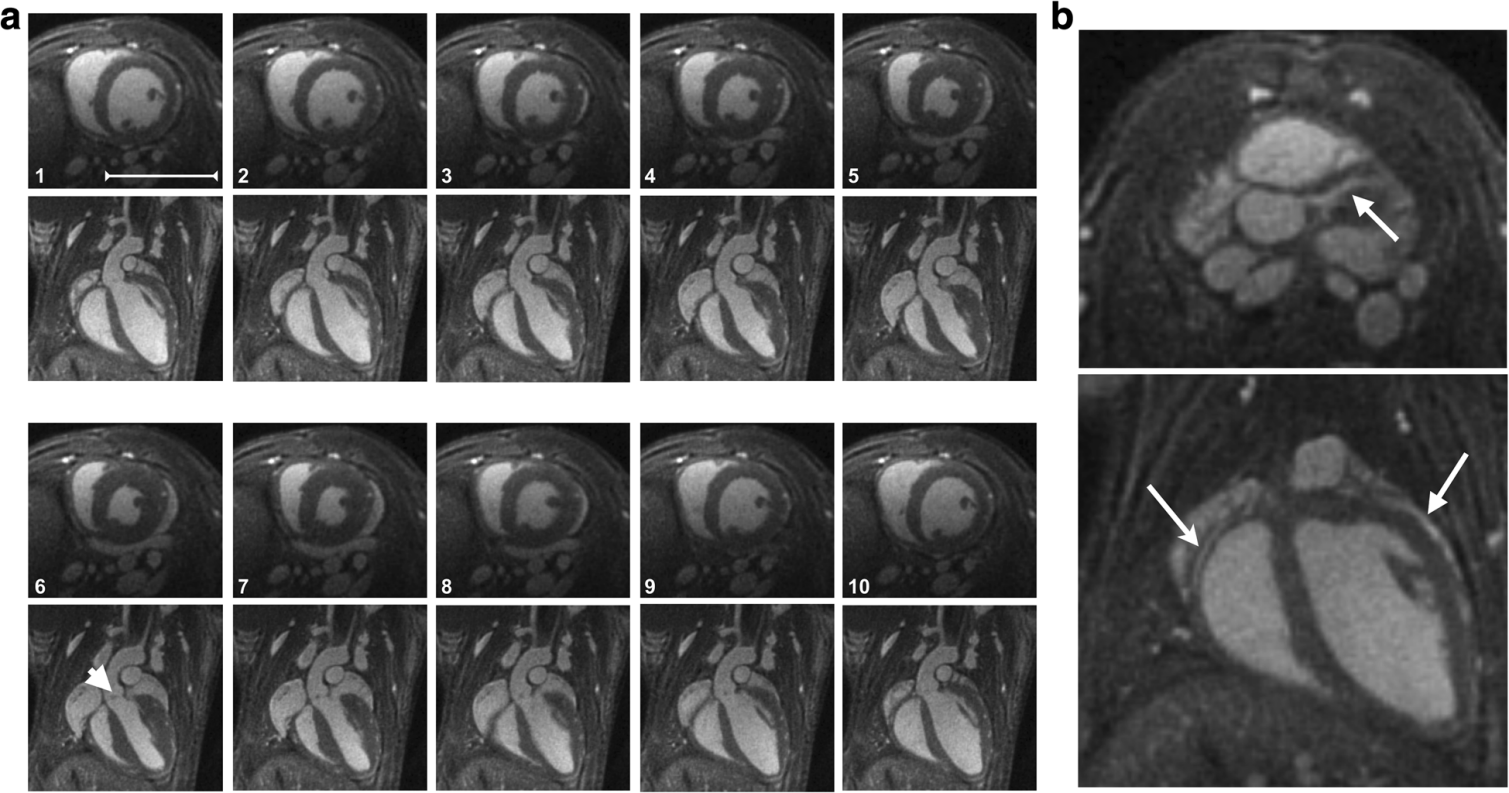
**a** 3D ECG-gated cine image as a function of cardiac cycle, obtained at 7 T at 104 μm isotropic resolution after injection of USPIO at 200 μmol Fe/kg. Short axis: upper panel; long axis: lower panel. No respiratory gating was used. The arrow (*image 6*) indicates the aortic valve. **b** Slices extracted from a 3D volume showing the coronary arteries (*right coronary arteries: arrow in upper image; right coronary arteries and left coronary arteries: arrows lower image*). The scale-bar represents 1 cm

Images at 104-μm isotropic resolution were acquired at 7 T (Fig. 5). Such a resolution necessitated 35 min acquisition. As expected, the concentration of contrast agent was sufficient during the experiment to enable a very good apparent signal-to-noise ratio for the blood (40.2 ± 2.3) and a good contrast-to-noise ratio between the blood and the myocardium (15.8 ± 2.0).

The spatial resolution appears to be effectively improved compared to images acquired at a resolution of 156 μm. This improvement in resolution can be used to perform precise volumetry (LVSV (μL) = 27.1 ± 3.2; LVEF (%) = 64.1 ± 4.2; RVSV (μL) = 27.4 ± 3.6; RVEF (%) = 61.8 ± 5.2).

It can also be used to better appreciate the deformations of the aortic arch during the cardiac cycle and to distinguish aortic valve (arrowheads, Fig. 5a N°6) and to track the left and right coronary arteries (arrows, Fig. 5b).

Movies showing the 4D-cine images are available as Additional file 1, Additional file 2, Additional file 3 and Additional file 4.

Finally, with the acquisition method used, although it decreases the spatial resolution and the signal-to-noise ratio due to 4 times less number of projections per cine, it is also possible to reconstruct a greater number of images per cardiac cycle. In the videos shown in Additional file 3 and Additional file 4, forty images were reconstructed per R-R interval.

## Discussion

This article demonstrates that by combining the use of a UTE 3D imaging sequence with USPIO-based contrast agents it is possible to achieve a strong Signal-to-Noise Ratio and a strong positive contrast at high magnetic fields (4.7 and 9.4 T), and thus to generate highly temporally and spatially resolved images of the cardiovascular system in small animals.

The term “apparent SNR” instead of SNR was used in this article because the method of SNR calculation did not take into account the spatial variance of noise of phased-array coils. However, as mentioned by Kellman et al. [30], this spatial variation is important only when parallel reconstruction is used which was not the case in our experiments.

We believe that the contrast obtained here is greater than those described to date in the literature on mouse cardiac MRI [31–34].

Up to now, iron nanoparticles have mainly been used through their T2* effect, which allows their presence to be detected at high magnetic fields (≥4.7 T), in particular in the field of cellular imaging. Recently, Strobel et al. [35] exploited the T1 effect of iron oxide particles to detect pulmonary inflammation. However, in these experiments, the increase in signal for the lung appears weak, probably due to a low concentration of contrast agent in the observed zone.

At 3 T, Girard et al. [24] showed that, with a subUTE sequence, it is possible to have a positive T1 effect and to exploit it to demonstrate tumor targeting *in vivo*. At lower magnetic fields (< 3 T) [6, 36, 37], and recently at 3 T [12, 13], iron nanoparticles have been shown to be good contrast agents for angiography with classical gradient echo sequences.

In these studies, to limit the susceptibility effect, contrast agent was injected at doses between 50 and 100 μmol Fe/kg. At high magnetic fields, as used here, a dose of 50 μmol Fe/kg gives a positive contrast with a UTE sequence. However, this contrast is lower than with other concentrations and decreases over time. It can therefore limit the acquisition of high-resolution images because a high contrast is necessary during more than 30 min.

With higher doses of injections, the contrast-to-noise ratio was significantly increased for all magnetic fields, and it remained constant for a duration compatible with high-resolution cardiac cine image acquisition (> 40 min). Although the doses injected in this study were higher than the doses used for clinical angiography, they remained significantly lower than the doses used for *in-vivo* targeting imaging (1000 μmol Fe/kg) [37].

Compared to gadolinium chelate-type contrast agents, the major advantage of iron nanoparticles is their much longer half-life in the blood, which permits 3D-cine acquisition in small animal. Other gadolinium-based contrast agents have been developed which have a longer half-life. For example, reports are given for p846 [6], a blood pool contrast agent consisting of a single gadolinium ion in a macrocyclic 3-armed chelate, developed by Guerbet (Aulnay-sous-bois, France), or gadolinium-loaded liposomes [31, 32]. However, none of these agents has been approved for clinical use, and their long-term innocuousness has yet to be confirmed. In contrast, a large variety of iron-based nanoparticles has been developed with various sizes, coating compounds and surface charges (Sinerem ®, Endorem ®, P904 ® from Guerbet, Resovist ® and Supravist ® from Bayer Schering, Clariscan ® from Amersham Health and Ferumoxytol ® from AMAG Pharmaceuticals, …) [38] and tested on small animals or humans. Among them, P904 is still available for preclinical imaging (Chematech, Dijon, France). Ferumoxitol has recently gained in interest in clinical MRI [39] due to its U.S. FDA approval as an iron supplement for patients with chronic kidney disease. Moreover, its higher relaxivity compared to Sinerem (r1 = 2 mM^−1^ s^−1^ at 7 T [25]) combined with a prolonged circulating half-life (14–15 h), should make it an excellent contrast agent for high field MRI.

Since such contrast agents are strongly paramagnetic, ultra-short echo times are needed to achieve significant positive contrast. The UTE sequence used in this study has several advantages. The use of radial encoding with sampling from the center of the k-space, combined with 3D imaging without slice selection and the use of a short excitation pulse (50 μs) makes it possible to generate extremely short TEs, less than 0.05 ms in this study. In small animals, this type of sequence has already been used for 2D-cine cardiac imaging and makes it possible to observe a time-of-flight effect. However, resolution of 2D MRI is often limited in the third dimension of space, and the TE is increased (> 0.3 ms), which can limit its usefulness when a contrast agent with a strong susceptibility effect is injected.

The other advantage of UTE sequences is their low sensitivity to several artefacts due to motion or blood flow. Thus, the signal from blood in areas particularly prone to turbulent flow appears quite homogeneous. This makes it possible to unambiguously measure the volume of the ventricles, whereas in conventional bright-blood images measurements can be affected by errors due to signal loss caused by significant phase-shifts of the spins in blood. The method presented here (UTE + USPIO) could thus be used for phase imaging in regions of extremely turbulent blood flow. Kadbi et al. [40] have already demonstrated the efficacy of UTE sequences to measure blood flow. However, to obtain a significant time-of-flight effect, they had to introduce a slice selection into the UTE sequence, thus increasing the value of TE. The method used here, using both non-selective volume excitation and USPIO injection, allows shorter TE and would be also useful for blood flow quantification.

Finally, we have shown that the combination of the 3D UTE sequence with the injection of an USPIO-type contrast agent makes it possible to generate very high spatial resolution, even though iron-based nanoparticles are known to diminish this resolution, particularly at high magnetic fields. Thus, by using significantly lower voxel sizes than those used to date in the literature [34, 41], a particularly precise ventricular volume can be determined (with semi-automated segmentation) in a relatively short acquisition time (around 12 min for a resolution of 156 μm, and around 35 min for a resolution of 104 μm). This should make it possible to study numerous models of heart diseases. In addition, the coronary arteries (right and left) are clearly visible at high resolution (Fig. 5b) as a function of the heart rate, whereas previous coronary images in small animals were acquired only during diastole [42, 43].

Among the limitations of the method proposed, the acquisition time for radial UTE images appears long compared to classical Cartesian methods, particularly when high spatial resolutions are necessary. However, as previously shown [44], radial methods are favorable to the use of compressed sensing reconstruction algorithms which can be used to limit the acquisition time.

One caveat of this study is that the doses of contrast agent used are higher than those commonly used for USPIO-based angiography in humans. However, they remain lower than those used in targeting imaging. In the absence of additional data, it is not possible today to use these doses as a first-pass agent for angiography in humans. However, doses between 50 and 100 μmol Fe/kg combined with 3D UTE imaging sequences, particularly at magnetic fields of 3 T or higher, should make it possible to image human vascular systems with temporal and spatial resolution which could be higher than those currently used in the clinical practice.

## Conclusion

In conclusion, we have demonstrated that by combining the injection of iron nanoparticles with 3D-cine UTE sequences, it was possible to generate a strong positive contrast between the blood and surrounding tissues. These signals and their high contrast were exploited to produce images of the cardiovascular system in small animals at high magnetic fields with a high spatial and temporal resolution. It can be useful for measuring the functional cardiac parameters or for assessing anatomical modifications to the blood vessels in disease models.

## Additional files

Additional file 1:
**Movie generated from the ten 3D cine images at 104 μm isotropic resolution.** Transverse orientation. The slices go from the aortic arch to the apex of the heart. Additional file 2:
**Movie generated from the ten 3D cine images at 104 μm isotropic resolution.** The slices go in the direction of the coronal plane. Additional file 3:
**Movie of the heart in the direction of the short axis.** The same data are used for the images at 104 μm isotropic resolution, but here, forty images per cycle were reconstructed with high temporal resolution encoding scheme. Additional file 4:
**Movie of the heart in the direction of the long axis.** The same data are used for the images at 104 μm isotropic resolution, but here, forty images per cycle were reconstructed with high temporal resolution encoding scheme.

## Abbreviations

UTE: Ultra-short echo time
FID: Free induction decay
ROI: Region-of-interest
TE: Echo time
TR: Repetition time
